# Mechanical Characterization and Modelling of Subcellular Components of Oocytes

**DOI:** 10.3390/mi13071087

**Published:** 2022-07-08

**Authors:** Yue Du, Yizhe Chen, Shuai Zhang, Dai Cheng, Yaowei Liu, Qili Zhao, Mingzhu Sun, Maosheng Cui, Xin Zhao

**Affiliations:** 1 The Tianjin Key Laboratory of Intelligent Robotics, Institute of Robotics and Automatic Information System, Nankai University, Tianjin 300350, China; duyue@nankai.edu.cn (Y.D.); 2120190357@mail.nankai.edu.cn (Y.C.); 2120190389@mail.nankai.edu.cn (S.Z.); 2120210429@mail.nankai.edu.cn (D.C.); liuyaowei@mail.nankai.edu.cn (Y.L.); zhaoqili@nankai.edu.cn (Q.Z.); sunmz@nankai.edu.cn (M.S.); 2 Institute of Intelligence Technology and Robotic Systems, Shenzhen Research Institute of Nankai University, Shenzhen 518083, China; tjsnykxyxmsyyjs@tj.gov.cn; 3 Institute of Animal Science and Veterinary of Tianjin, Tianjin 300381, China

**Keywords:** oocyte, zona pellucida, cytoplasm, AFM indentation, force clamping, viscoelastic model

## Abstract

The early steps of embryogenesis are controlled exclusively by the quality of oocyte that linked closely to its mechanical properties. The mechanical properties of an oocyte were commonly characterized by assuming it was homogeneous such that the result deviated significantly from the true fact that it was composed of subcellular components. In this work, we accessed and characterized the subcellular components of the oocytes and developed a layered high-fidelity finite element model for describing the viscoelastic responses of an oocyte under loading. The zona pellucida (ZP) and cytoplasm were isolated from an oocyte using an in-house robotic micromanipulation platform and placed on AFM to separately characterizing their mechanical profiling by analyzing the creep behavior with the force clamping technique. The spring and damping parameters of a Kelvin–Voigt model were derived by fitting the creeping curve to the model, which were used to define the shear relaxation modulus and relaxation time of ZP or cytoplasm in the ZP and cytoplasm model. In the micropipette aspiration experiment, the model was accurate sufficiently to deliver the time-varying aspiration depth of the oocytes under the step negative pressure of a micropipette. In the micropipette microinjection experiment, the model accurately described the intracellular strain introduced by the penetration. The developed oocyte FEM model has implications for further investigating the viscoelastic responses of the oocytes under different loading settings.

## 1. Introduction

The early steps of embryogenesis are controlled exclusively by the quality of oocyte. The elasticity of ZP outer layer can be used as a marker for the quality of the oocyte, which decreases from immature MI to good-quality MII, up to poor-quality MII oocytes [1,2]. At the time of fertilization, those oocytes that are overly stiff fail to release cortical granules properly, while insufficient zona hardening will occur for the oocytes that are overly soft after fertilization. The ZP is a glycoprotein layer that surrounds the plasma membrane of oocytes. It is composed of glycosylated proteins that are synthesized and assembled into cross-linked fibrils [3]. Oocytes are rich in the cytoplasm, which exhibits high heterogeneity, which arises from the presence of densely clustered cross-linked filaments, nucleus and organelles. Ideally, a compelling accurate cell model is expected to describe the roles of different components. However, a majority of studies regarding the mechanical properties of oocytes have assumed that it is homogeneous [1,4], partly due to the technical difficulty in mechanical characterization of the subcellular structures. The limited documents of ZP and cytoplasm show a disparity in Young’s modulus of ZP and the viscosity of the cytoplasm.

Specifically, it is widely suggested that the glycoproteins filaments of ZP are cross-linked by non-covalent bonds, so it has been modeled as an incompressible elastic material [5]. The Young’s modulus has been estimated to range from 7.4 kPa to 36.9 kPa in mammalian oocytes [6,7,8,9]. This assumption of pure elasticity was extended by the work in [10,11], which reported the experimental results manifesting the viscoelastic behavior of ZP. The cytoplasm has been assumed to behave as a viscous, non-Newtonian fluid with the reported viscosity spanning several orders of magnitudes less than 0.1 Pa·s to $4\times 10^{3}$ Pa·s for a wide range of cells [12,13,14,15,16,17]. On the one hand, this could originate from the measurement method, the result from dealing with the surface coated magnetic beads manipulated with a magnetic field was quite different from that of tracking intracellular vesicles or nanoparticles dispersed in cytoplasm [17,18]; on the other hand, it could be because that the cytoplasm exhibits the high heterogeneity arising from the presence of filaments, nucleus and organelles. The cytoplasm was also suggested to behave as a poroelastic material at a short time scale, but this assumption failed to describe the rheological behavior of the cytoplasm observed over a wide range of timescales [19]. Considering the generalized uncertainty, it may still be reasonable to assume that the cytoplasm has viscoelastic properties, the values of which are further needed to be determined through a standard testing protocol. The reported values of Young’s modulus of cytoplasm ranged from 0.015 kPa to 13 kPa [14,17].

Many experimental apparatuses and approaches for characterizing a cell’s mechanical responses are global methods, meaning that they focused on the entire cell instead of subcellular structures. Micropipette aspiration is a classical way of measuring the time-dependent response of cells by recording the aspiration rate at a given pressure and the retraction rate, but it can only measure the mechanical properties of a cell as a whole structure [20,21,22]. A dedicated model needs to be developed in order to separate the effects of ZP and cytoplasm, but this requires knowledge about the mechanical properties of these subcellular structures. Magnetic tweezers work by attaching the beads to the target that is commonly the cell surface, and then imposing a periodic magnetic field on the bead to analyze the movement of bead [23,24]. The microfluidic chips have been designed for indentation and cell squeezing tests, but it is too complicated for them to access the cell interior [25,26]. A solution to this problem can be the use of the AFM that provides the localized measurement with high spatial and force resolution. AFM enables the observation of the viscoelastic responses of subcellular components if the probe can access to them [27,28,29]. With the properties of each component, a compelling cell model is expected to assemble each component properly to reflect the mechanical response of the cell as a whole structure.

In this work, we developed a high-fidelity oocyte FEM model that took the separate roles of ZP and cytoplasm into consideration. To do it, we first characterized the mechanical profiling of the ZP and cytoplasm using the force clamping technique in the AFM creep test. While the force was clamping, the viscoelastic creep behavior featuring an exponential decay in the deformation rate was fitted to the Kelvin–Voigt model to derive the model parameters. We analyzed the results from multiple cells and different measurement locations to estimate the variance. The shear relaxation modulus and relaxation time were separately determined for the ZP and cytoplasm by providing the stress relaxation data generated from the Kelvin–Voigt model to the Proxy Shear Relaxation module of the ANSYS. Three FEM oocyte models, namely the ZP and cytoplasm model, the isolated ZP and cytoplasm model and the homogeneous model were developed to investigate the separate effects of ZP and cytoplasm on the viscoelastic properties of oocytes. The ZP and cytoplasm model took the properties of the ZP that was not isolated from the oocyte. For comparison purposes, we built the homogeneous model as the whole-cell model and the isolated ZP and cytoplasm model using the mechanical profiling of ZP debris isolated from the oocyte for the ZP layer. The performance of the models was firstly examined by comparing the dynamic aspiration process observed in the micropipette aspiration experiment and its simulation under varying experimental conditions. Then, they were validated in the micropipette penetration experiment by comparing the penetration-induced intracellular strain between the experiment and the simulation. The organization of the paper is as follows. Section 2 summarizes the materials and methods used in this study involving the isolation of the ZP and cytoplasm from an oocyte through the micromanipulation, the AFM setup for measuring the viscoelastic properties of the ZP and cytoplasm, the development of the FEM oocyte models and the simulation settings of the micropipette aspiration and penetration experiments. Section 3 presents the results regarding (i) the characterization of the viscoelastic properties of cytoplasm, the isolated ZP and the whole cell, (ii) validation of the models in describing the time-varying aspiration depth of an oocyte under a step negative pressure and (iii) validation of the models in analyzing the intracellular strain induced by the micropipette penetration. Section 4 and Section 5 discuss and conclude the study.

## 2. Materials and Methods

### 2.1. Preparation for Mechanical Characterization of Subcellular Components

We used ovaries acquired from a slaughterhouse and delivered to our laboratory within 2 h of collection in a thermos flask containing sterile saline at 35–37 ${}^{\circ }$C. The experiment was reviewed and approved by the Animal Care and Use Committee of Nankai University on 15 March 2015. The cumulus oocyte complexes (COCs) with a diameter of 2–6 mm were collected from follicles with a disposable syringe, and only those with uniform ooplasm and compact cumulus cells were cultured in a maturation medium in an incubator with 5% CO${}_{2}$ and 95% humidified air atmosphere. The maturation medium consisted of TCM199 (with Earle’s Salts; Gibco, Grand Island, NY, USA) supplemented with 10% porcine follicular fluid (PFF), 0.1 mg/mL cysteine, 0.065 mg/mL penicillin, 10 ng/mL epidermal growth factor (EGF), 10 IU/mL equine chorionic gonadotropin (eCG; Intervet Pty. Ltd., Boxrneer, Australia) and 10 IU/mL human chorionic gonadotrophin (hCG; Intervet Pty. Ltd.). Survival oocytes showed homogeneous cytoplasm and intact, bright membrane under a stereomicroscope, and those with the first polar body being expelled into the perivitelline space represented the matured oocytes.

**Figure 1:**
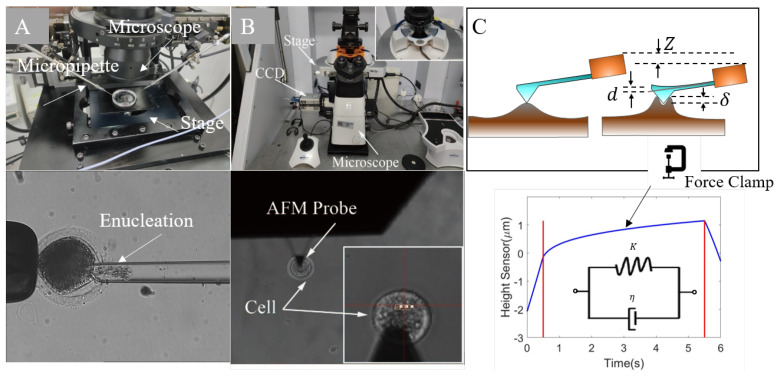
The AFM measurements. (**A**) An in-house robotic platform (upper image) designed for cell micromanipulation (injection, rotation, enucleation, etc.) was employed to isolate the ZP and cytoplasm from an oocyte. It integrated an inverted microscope, a pair of customly designed X-Y-Z micromanipulator, X-Y stage, micropipette holders, a CCD camera, a pressure controller, etc. We maneuvered the micropipette to aspirate out cytoplasm (enucleation, bottom image) and leave the ZP debris. Both the cytoplasm ball and the ZP debris were immediately transferred to the polylysine-coated slide for measurement. (**B**) Bruker Bioscope Resolve AFM (upper image) integrating baseplate, CCD camera, inverted optical microscope, probe holder, SPM head and EasyAlign unit. During measurement, the indentation was performed on the multiple sites of an specimen, as shown in the bottom image. (**C**) The calculation of indentation from the scanner movement and cantilever deflection δ=Z−(1+e)d, *e* is the calibration error of the deflection sensitivity. The force was clamped for several seconds once the predefined value was reached to observe the viscoelastic behavior of the oocyte and derive the parameters for the Kelvin–Voigt model.

Instead of using a long, sharp tip to consequently penetrate the subcellular structures, we herein used an in-house robotic platform (Figure 1A designed for cell micromanipulation (injection, rotation, enucleation, etc. [30,31]) to isolate the ZP and cytoplasm from an oocyte. The AFM measurements were performed right after the isolation. The robotic platform that we used integrated an inverted microscope (Olympus BX-51), a pair of customly designed motorized X-Y-Z micromanipulators with the range of 50 mm × 50 mm × 50 mm and repeatability of 1 μm, an X-Y stage with the range of 100 mm × 100 mm and repeatability of 1 μm, three micropipette holders (Narishige, HI-7, Setagaya, Japan), a double pipette holder (Narshige, HD-21, Japan), a CCD camera (Panasonic W-V-460), a customly designed syringe, a pressure controller, a pneumatic pump and a host computer. We used the following experimental procedures to readily isolate ZP and cytoplasm. To obtain the cytoplasm, a micropipette with inclined incision was maneuvered to penetrate the cell and aspirate out a portion of the cytoplasm. The cytoplasm formed a ball after being placed into the PBS solution. The cytoplasm ball was transferred to the polylysine-coated slide, which allowed for the adhesion of the cytoplasm to the slide surface. To acquire the ZP debris, we squeezed the cell using a flat micropipette causing its rupture. We increased the pressure in the micropipette to completely aspirate out the cytoplasm, which left the ZP to be transferred to the polylysine-coated slide. The AFM measurements were performed immediately after the preparation of the cytoplasm or ZP specimen was completed.

### 2.2. The Setup of AFM Measurements

The AFM experiment was carried out on a Bioscope Resolve AFM (Bruker, Billerica, MA, USA) integrating an inverted optical microscope (Nikon, Eclipse Ti2, Japan), a CCD camera, an SPM head that was a detector-probe assembly for reproducible positioning of the probe and a baseplate with XY scan stage and mounted sample stage (Figure 1B). The probe was MLCT-BIO-DC A designed for imaging in fluid as well as force spectroscopy of biological samples. The geometrical design of the probe helped to increase the success rate of the force pulling experiments. Enhancing the probe with a gold pad helped to compensate the drift of the probe. The used parameters of the probe were as follows. Geometrically, it had a triangular shape that with a propriety design to avoid bending the probe. It had an approximate tip radius of 20 nm, and an approximate length of 175 μm. The spring constant was 0.07 N/m, and the width was 22 μm. The AFM indentation was performed on the oocytes (ZP or cytoplasm) placed on a polylysine-coated slide for mounting and immersed in PBS solution. The imaging mode was PeakForce QNM in fluid, which directly controlled the peak normal force and minimized the lateral force on the probe. The peak force setpoint was 12.67 pN, and the frequency was 1 kHZ. Before the experiment, the spring constant of the probe was calibrated using the AFM thermal noise module. The AFM probe was used to detect the Young’s Modulus of the oocytes (ZP and cytoplasm) through the approach-retraction mode. In the approach stage, the probe was navigated to arrive at the central region of the oocyte and gradually approach its surface with the help of the optical microscope. Once the probe contacted the cell surface, the indentation took place, and the cantilever deflected (deflection [nm] = deflection [volts]/sensitivity) to reflect the force (spring constant × deflection [nm]) exerted on the probe. The retraction stage began as the deflection or the ramp size reached the specified value (trigger threshold = 12.45 nm, ramp size = 2 μm) with the probe being lifted away from the cell. We recorded the force-indentation data to derive the Young’s Modulus of the oocyte such that multiple indentation sites in the central region were selected for each cell to eliminate the impact of heterogeneity on the results. In order to capture the dynamic viscoelastic response of the oocyte given a constant force, we indented the surface of the cell to reach the specified indentation force. Then, we held the force for a few seconds to record the height sensor vs. time curve, referred to as force clamping. More details can be found in the subsection of force clamping for viscoelastic creep behavior.

The force-indentation curves were fitted to the Sneddon model concerning the tip shape and Poisson’s ratio to derive the Young’s Modulus of the sample, expressed as follows:

(1) $$F=\frac{2}{\pi }\frac{E\delta^{2}}{\left(1-\upsilon^{2}\right)}\tan\alpha$$

where *E* denotes the Young’s modulus, υ=0.5 is Poisson’s ratio for the ZP or cytoplasm, and $\delta =18^{\circ }$ is the half angle of the tip.

Once the experiment started, the cantilever was approached to the cell at a constant velocity. Then, after the tip and the cell were brought into contact, the force on the cantilever was increased, and the indentation was stopped at a predefined value of the indenting force. Instead of retracting, the force was clamped at this value using a feedback loop controlling the z position of the cantilever. The viscoelastic creep of the cell was observed while the force was active. Dynamic viscoelastic parameters were extracted from the creep behavior measured during the force clamping phase. We used the Kelvin–Voigt model, which consists of a Newtonian damper and Hookean elastic spring connected in parallel, to describe the observed viscoelastic creep behavior,

(2) $$\delta \left(t\right)=\delta_{0}+\frac{F_{\mathrm{clamp}\;}}{k}\left[1-\exp\left(-\frac{k}{\eta }t\right)\right]$$

where $F_{\mathrm{clamp}\;}$ is the predefined force at which force clamping starts, *k* corresponds to the elastic component, η denotes the viscous component, and $\delta_{0}$ is the instantaneous elastic deformation of the sample.

### 2.3. The FEM Oocyte Model

**Figure 2:**
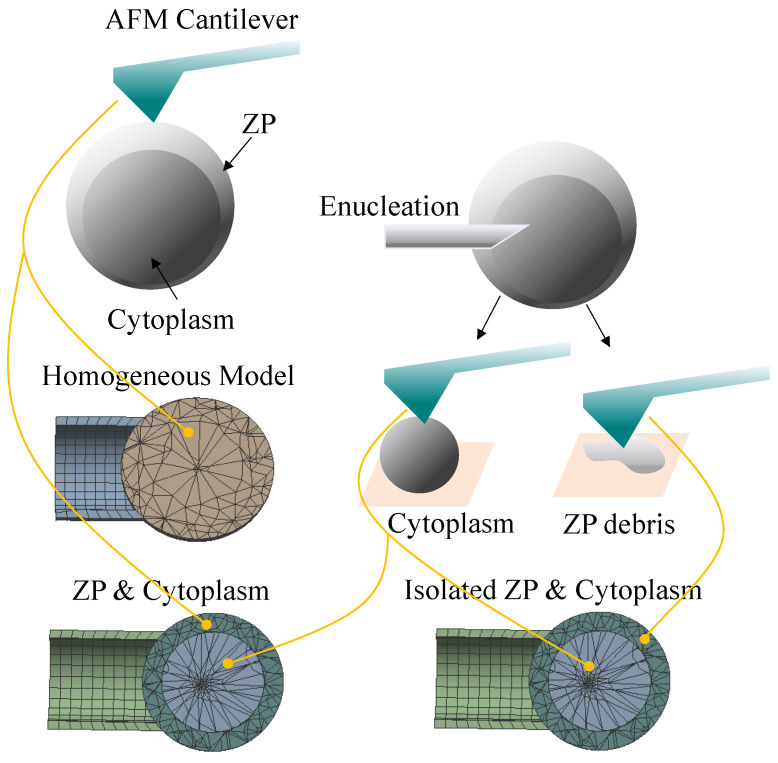
Three viscoelastic FEM models of oocytes that consider the roles of ZP and cytoplasm. The ZP and cytoplasm model has a layered structure mimicking its physical structure observed under a microscope. The ZP layer surrounded the cytoplasmic layer with the two layers bonded (no slip or separation). For comparison purposes, we built the homogeneous model as the whole-cell model and the isolated ZP and cytoplasm model using the mechanical profiling of ZP debris isolated from the oocyte for ZP layer. The viscoelastic properties of the intact ZP and the isolated ZP debris, i.e., shear relaxation modulus and relaxation time, were derived in the AFM indentation and viscoelastic creep test. The same experimental tests were performed to analyze the cytoplasm that was separated from the oocyte through robotic enucleation.

We used a Kelvin–Voigt model to describe the creep behavior of the ZP and cytoplasm specimen. The explicit formula of the relationship between $F_{clamp}$ and indentation δ shown in Equation (2) was used to generate the stress relaxation data and provide it to the “Shear data-viscoelastic” module of ANSYS to derive shear relaxation modulus and relaxation time, which gave the viscoelastic properties of materials. Given the properties of the ZP and cytoplasm, we developed an oocyte model that considered the separate effects of the two subcellular components on the viscoelastic responses of the oocyte upon loading. The model has a layered structure mimicking its physical structure observed under a microscope. The ZP layer surrounded the cytoplasmic layer with the two layers bonded, meaning that no slip or separation was allowed for the two. For comparison, we built an isolated ZP and cytoplasm model that took the properties of the ZP debris which had been isolated from the oocyte. Furthermore, we developed a homogeneous model as a whole-cell model with the assumption that the cell was isotropic and homogeneous. The viscoelastic parameters of the homogeneous cell model were set according to the creep behavior of the intact ZP due to the fact that ZP wrapping up the cytoplasm was much more rigid than the cytoplasm. The ZP’s creeping curve that was described by the relationship $F_{clamp}$ and indentation δ was used to derive shear relaxation modulus and relaxation time in ANSYS for setting the homogeneous model. The graphic illustration of how the three models were formed is given in Figure 2. The three models were defined in the ANSYS Workbench 2020 R2.

### 2.4. Validation Experiment I—Micropipette Aspiration Experiment

#### 2.4.1. The Simulation Settings for Micropipette Aspiration

When developing the model in the ANSYS, we assumed that the ZP had a uniform thickness, and the cytoplasm had a spherical body. We statistically measured the radius of 100 oocytes and their ZP thickness, and found the average radius of the oocyte to be 80 ± 5 μm and its average ZP thickness to be 20 ± 5 μm. Therefore, we set the cell radius to 75 μm, the ZP thickness to 20 μm, and the cytoplasm radius to 55 μm in the simulation. During model validation, the cell radius varied from 75 μm to 85 μm and the ZP thickness varied from 15 μm to 25 μm. The wall of the micropipette was set to 1/10 of its inner diameter 80–90 μm. All the three oocyte models were built with a 3D axisymmetric FEM scheme in ANSYS environment. The SOLID187 element was employed for modeling ZP and cytoplasm, while the SOLID186 element was utilized for the micropipette. Both elements have plasticity, hyperelasticity, creep, stress stiffening, large deflection and large strain capabilities. The calculation was performed based on the assumption that a large deformation would occur. We set the Poisson’s ratio for the ZP and cytoplasm to 0.5 and for the glass pipette to 0.25. Under nearly incompressible conditions (Poisson’s ratio is close to 0.5), any small error in the predicted volumetric strain will result in a large error in the stresses. This error will in turn influence the displacement prediction and may generate displacements very much smaller than they should be for a given mesh. To over this difficulty, the mixed u-P formulations were used in ANSYS where the volume change rate was interpolated on the element level and solved on the global level independently in the same way as displacements such that the solution accuracy was independent of Poisson’s ratio. The contact mode between the micropipette and ZP was defined to be frictional with a friction coefficient of 0.2, while that for the ZP and cytoplasm was bonded. In the simulation of micropipette aspiration, we fixed the micropipette and imposed a negative pressure perpendicular to the deformed surface of the cell. A convergence study (type: maximum, allowable change: 50%, max refinement loops: 6) was carried out to verify the convergence when calculating total deformation in the micropipette aspiration experiment. The static structural analysis mode with a step time of 0.05 s was used.

#### 2.4.2. The Subpixel Detection of Cell Edge for Micropipette Aspiration

The cell model was validated in the aspiration experiment by comparing the simulated change of aspiration length with time with the true process. During the aspiration experiment, an oocyte was deposited next to a micropipette with a radius falling into [30, 50 μm]. A step negative pressure within [−13 hPa, −50 hPa] was applied to slowly suck a portion of the cell into the micropipette. The cell edge at each frame (30 frames/s) was detected with subpixel precision to compute the aspiration depth.

In the aspiration experiment, the rate of acquiring images was low and the change of the aspiration length with time was subtle; therefore, using the pixel-level cell edge detection methods would introduce large errors. We used the subpixel-level detection method based on the first three gray moments of the image, which were computed using a 7 × 7 kernel for the image convolution operation. The edge was detected if the following requirements were met: $|H_{2}-H_{1}|>2\lambda$, λ>τ and $\rho \leq \frac{2}{N}\alpha$, where ρ represents the vertical distance of the edge from the kernel center; $H_{1},H_{2},\lambda$ denote the parameters calculated from the first three gray moments; τ and α represent adjustable variables. Please see the supplemental materials for the details about the determination of those parameters. The coordinates of the detected edge were given as follows:

(3) $$\left[\begin{array}{c} x_{e}\\ y_{e} \end{array}\right]=\left[\begin{array}{c} x\\ y \end{array}\right]+\frac{N}{2}\rho \left[\begin{array}{c} \cos\theta \\ \sin\theta \end{array}\right]$$

where (x,y) denotes the center of the weight kernel, *N* is the size of the kernel, θ is the angle between the normal vector and horizontal axis.

### 2.5. Validation Experiment II—Microinjection Experiment

#### 2.5.1. The Simulation Settings for Microinjection Experiment

To further validate the performance of the FEM oocyte model, we simulated the microinjection experiment and calculated the intracellular normal strain along the axis aligning with the microinjection direction to compare it to the experimental results. To recover the real microinjecting process, we recorded the displacement of the microinjector tip from the start of microinjection to the time when the ZP membrane was penetrated through during the experiment and set the value to the simulated microinjector. The setting of the microaspiration pipette and FEM model were the same as that in the simulation of the microaspiration experiment. The diameter of the microinjector was 20 μm and the inclination tip angle was $45^{\circ }$.

#### 2.5.2. Detection of Microinjection-Induced Intracellular Strain from Microscopic Images

We used the modified Farnebäck optical flow algorithm to detect the intracellular strain from the time-lapse microscopic images of the oocyte subjected to the microinjection. The details were documented in our previous studies [32,33]. The luminance of each pixel point was fitted to a quadratic polynomial model. The displacement of the pixel point during the two frames was formulated by assuming the luminance of the neighboring region of a pixel point stayed unchanged during motion. In our method, it was modified using a deformation matrix *K* to increase the precision of calculating the pixel displacement by considering the continuity of material.

(4) $$v\left(x\right)=\min_{v(x)}\sum_{\Delta x\in I}\omega \left(\Delta x\right)||A\left(x+\Delta x\right)Kv\left(x\right)-{\Delta b\left(x+\Delta x\right)||}^{2}$$

The element of *K* in *x* direction was ${\bar{v}}_{x}\left(x+\Delta x\right)/{\bar{v}}_{x}\left(x\right)$, describing the relative displacement between a neighboring pixel point and the target one. This value was calculated from the fitted quadratic polynomial model based on the microinjection-induced displacement data.

## 3. Experimental and Simulation Results

### 3.1. The Mechanical Characterization of the Subcellular Components of Oocytes

#### 3.1.1. The Viscoelastic Properties of the Oocyte Cytoplasm

**Figure 3:**
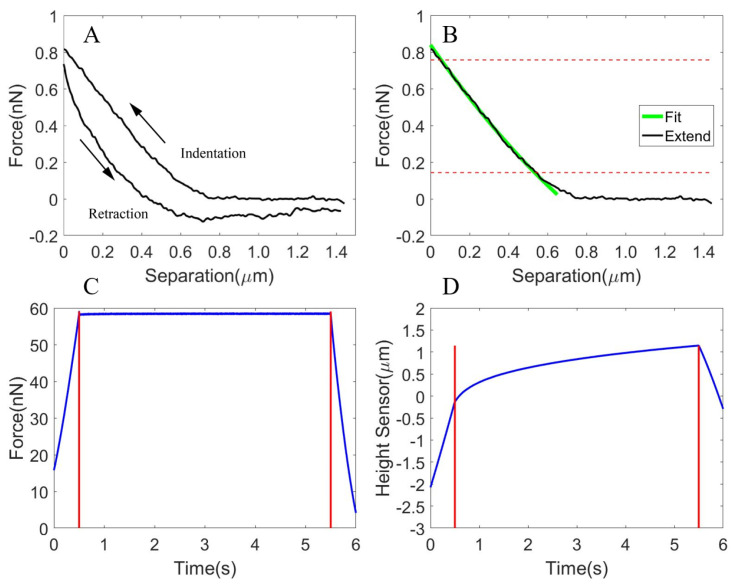
The response of the cytoplasm during AFM indentation for the development of three viscoelastic FEM oocyte models. (**A**) The force-indentation curve (indentation and retraction processes). The hysteresis area, i.e., the area enclosed by the indentation and retraction curves was observed. (**B**) The fitting of the indentation (extending process) curve using the Sneddon model (focusing on the portion with a large slope between the two red horizontal lines). (**C**) The clamping of force at the predefined value for 5 s. (**D**) The viscoelastic creep curve.

The Young’s Modulus of the oocyte cytoplasm was measured by gradually pressing the AFM probe onto the cytoplasm, which formed a spherical body after being extracted from the oocyte. Once the tip was brought into contact with the cytoplasm, the force on the cantilever started to increase, as shown by the indentation curve in Figure 3A. The tip started to retract when either the safe threshold force or the ramp size (set to 2 μm) was reached. By fitting the force-indentation curve (using the portion with the large slope as shown in Figure 3B) with the Sneddon model, the average Young’s Modulus of the cytoplasm for multiple indentation sites was 3.16 ± 0.35 kPa (0.75 kPa [34] for mouse oocyte cytoplasm). The indentation and retraction were hysteretic and did not align with each other, as shown in Figure 3A, which was due to the hysteresis effect of the scanner.

Using the force clamping technique, the force was clamped at the predefined value through a feedback loop controlling the z position of the cantilever. We observed the viscoelastic creep behavior of the cytoplasm and derived the parameters of the spring and dashpot of the Kelvin Voigt model fitted to the creep curve. Figure 3C shows that the force was held for 5 s after it reached nearly 60 nN, and Figure 3D shows the recorded viscoelastic creep curve. The parameters of the spring and dashpot were measured to be k=0.052±0.005 N/m and η=0.082±0.009 N·s/m.

#### 3.1.2. The Viscoelastic Properties of the Isolated ZP

**Figure 4:**
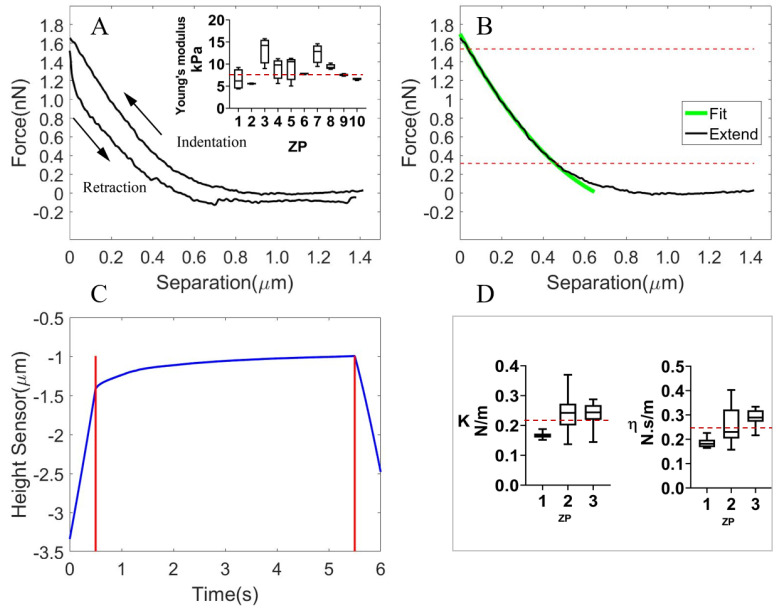
The response of the isolated ZP during AFM indentation for the development of the isolated ZP and cytoplasm model. (**A**) The force-indentation curve (indentation and retraction processes). The included figure shows the estimated Young’s Modulus of multiple ZP lumps. The average value is indicated with the red dotted line. (**B**) The fitting of the indentation (extending process) curve using the Sneddon model (focusing on the portion with a large slope between the two red horizontal lines). (**C**) The viscoelastic creep curve obtained by holding the indenting force at nearly 50 nN for 5 s. (**D**) The *k* and η values of the Kelvin–Voigt model that were used to describe the viscoelastic creep behavior of the isolated ZP.

During the experiment, we indented the isolated ZP, which was prepared by completely aspirating out the cytoplasm from an oocyte and transferring the debris to the polylysine-coated slide. The plotted force-indentation curve and fitted curve using the Sneddon model are shown in Figure 4A,B, respectively. The average Young’s Modulus of the multiple ZP debris (with different indentation sites for each) was estimated to be 8.77±2.38 kPa (22 kPa [35] for the isolated ZP from the mature bovine oocyte), shown in the included figure of Figure 4A. The variation of the measured value for different ZP debris and different indentation sites indicated the heterogeneity of the ZP structure. Using the force clamping technique, we observed the viscoelastic creep behavior (Figure 4C) of ZP debris and fitted the creep curve with the Kelvin model. The average *k* of the spring was estimated to be k=0.216±0.034 N/m, while the average η of dashpot was estimated to be η=0.245±0.044 N·s/m, as shown in Figure 4D.

#### 3.1.3. The Viscoelastic Properties of the Whole Cell

**Figure 5:**
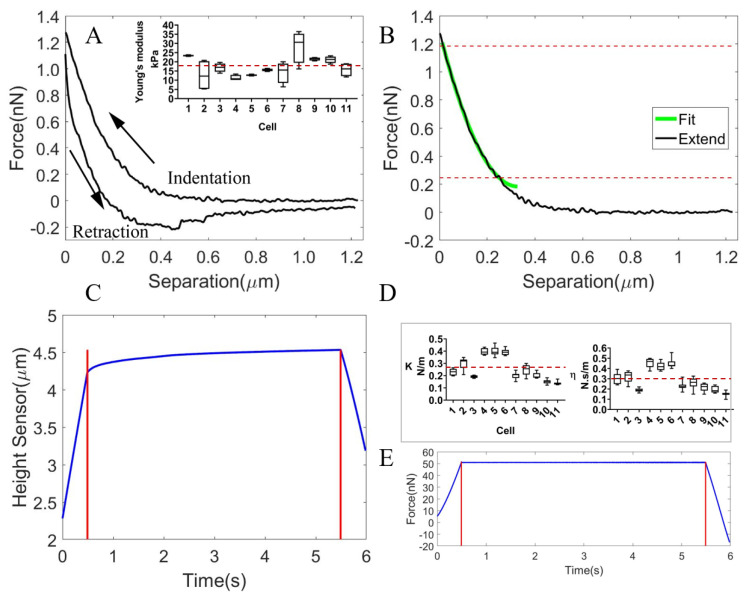
The response of the whole cell obtained by indenting the ZP surface using AFM for the development of the homogeneous model. (**A**) The force-indentation curve (indentation and retraction processes). The included figure shows the estimated Young’s Modulus of multiple cells. The average value is indicated with the red dotted line. (**B**) The fitting of the indentation (extending process) curve using the Sneddon model (focusing on the portion with a large slope between the two red horizontal lines). (**C**) The viscoelastic creep curve obtained by holding the indenting force at nearly 50 nN for 5 s shown in (**E**). (**D**) The *k* and η values of the Kelvin–Voigt model that was used to describe the viscoelastic creep behavior of the cell.

We indented the surface (ZP surface) of the whole cell and plotted the force-indentation curve, as shown in Figure 5A. Figure 5B shows the fitting curve that was generated using the portion with the large slope. A total of 10 matured oocytes were measured, each with multiple indentation sites. The average Young’s Modulus of the whole cell was 17.63±5.10 kPa (8.26–22.8 kPa [7,36] for mouse, porcine oocytes). Since the indentation was too small to reach the region beyond the ZP with a thickness of 15 μm [37], we actually indented on the ZP surface. Due to the volume effects, a moderately larger value was obtained compared with that of the isolated ZP debris. We observed that Young’s Modulus calculated from the AFM indentation data was about 20% smaller than that obtained through micropipette aspiration [36]. This was probably due to the effect of the measurement scale. The variation of the Young’s Modulus for the different indentation sites on the cell was profound, showing the heterogeneity of the ZP structure, which was composed of a network of cross-linked fibrils. Using the force clamping technique (Figure 5E), we analyzed the viscoelastic creep behavior (Figure 5C) of multiple cells and fitted it with the Kelvin model. The average *k* of the spring was estimated to be k=0.259±0.093, while the average η of the dashpot was estimated to be η=0.290±0.105 N·s/m. From Figure 5D, we could observe the variations of *k* and η for different cells as well as the similar tendency of such variations.

### 3.2. Validation of the Cell Model in the Micropipette Aspiration Experiment

**Figure 6:**
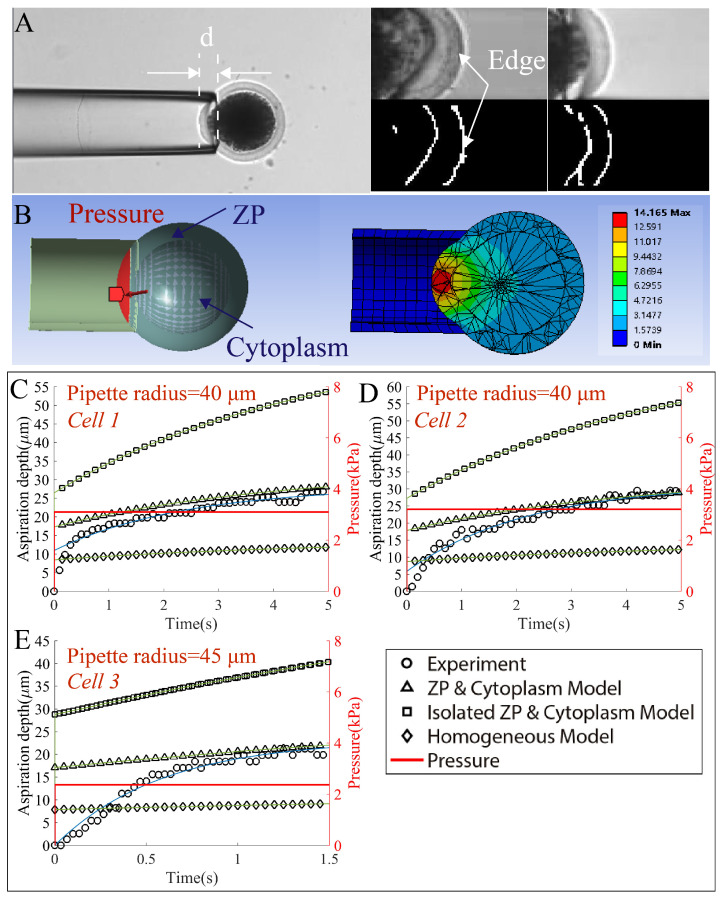
Performance comparison between the simulation and experimental results obtained from the micropipette aspiration experiment. (**A**) The aspiration of an oocyte using a micropipette on an in-house robotic micromanipulation platform. The cell edge was detected with a subpixel precision to compute the aspiration depth *d*. (**B**) The layered ZP and cytoplasm model. Given the pressure (indicated in red), the portion of cell gradually flowed into the micropipette. (**C**–**E**) Comparison of the experimental results (circle) with the simulation results of the three models (ZP and cytoplasm: triangle; isolated ZP and cytoplasm: square; homogeneous model: diamond) under the different settings of micropipette radius and pressure. Please note a step pressure was difficult to produce in the experiment; therefore, the instantaneous elastic deformation in response to the step pressure was not obvious for the experimental results. For the simulation results, the viscoelastic creep behavior was least obvious for the homogeneous model with the smallest instantaneous elastic deformation. In contrast, the isolated ZP and cytoplasm model was too soft and produced deformation that significantly deviated from the experimental data. It can be seen from all the three figures that the ZP and cytoplasm generated deformation that was in good agreement with the experimental data under different conditions.

A summary of the simulation parameters is shown in Table 1. For the setting of viscoelastic properties in ANSYS, The average relative modulus was 0.08 ($G_{i}/G_{0}$, *i* is the Proxy term and $G_{0}$ is the instantaneous shear modulus) and the relaxation time was 3.03 s for the ZP, while that for the cytoplasm are 0.278 and 1.297 s. Figure 6 demonstrates the comparison between the experimental observations of the change of aspiration length with time and that from the simulation using the three models, namely the ZP and cytoplasm, the isolated ZP and cytoplasm and the homogeneous model. An illustration of the experimental scene was shown in Figure 6A. As can be seen from the image, the subpixel detection of cell edge was achieved to calculate the aspiration depth. Figure 6B shows the oocyte model we developed in ANSYS, which had a layered structure with the cytoplasm layer surrounded by the ZP layer. The two layers were bonded to avoid relative movement or slipperiness. We placed the cell next to the micropipette in the simulation and imposed fractional constraints between the micropipette and the cell, and then exerted negative pressure to gradually suck the portion of cell into the micropipette. The intracellular deformation of the cell was shown in the image. We can see that the largest deformation occurred in the region near the micropipette tip. To observe the dynamic aspiration process, we tracked the aspiration length at each frame for both the experiment and simulation and plotted in Figure 6C–E for different combinations of the micropipette radius and pressure. We did not observe the same instantaneous elastic deformation of the experimental data as the simulation data, partially due to the presence of capillary pressure that gradually aspirated the cell into the micropipette and the difficulty in exerting a strict step pressure during the experiment. The three models exhibited the distinguishable viscoelastic behavior. Specifically, the viscoelastic creep behavior was least obvious for the homogeneous model that also generated the smallest instantaneous elastic deformation. In contrast, the isolated ZP and cytoplasm model was too soft and produced an apparently large deformation that significantly deviated from the experimental data. Only the ZP and cytoplasm generated deformation that was in good agreement with the experimental data under different combinations of micropipette radius and pressure with 5.39%±3.42% of discrepancy in the final aspiration length, which also accounted for the variance among different cells. Although the homogeneous model and ZP and cytoplasm model had the same properties of ZP, they exhibited different viscoelastic behavior, which warrants the separate modeling of the ZP and cytoplasm. This was further verified by the result that the ZP and cytoplasm agreed best with the experimental results.

**Figure 7:**
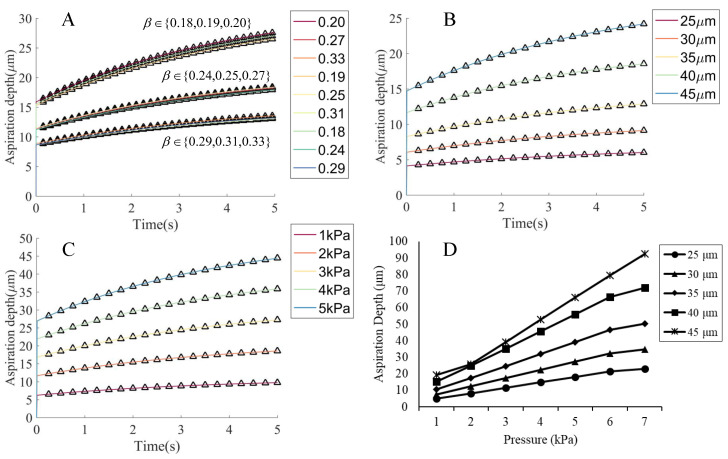
The effects of the ZP thickness, the radius of micropipette, and the aspiration pressure on the aspiration length in the simulation with the ZP and cytoplasm model. (**A**) The variation in the ZP thickness. We defined the parameter β=h/R, where *h* = 15, 20 or 25 μm and *R* = 75, 80 or 85 μm. The β varied from 0.18 (15/85) to 0.33 (25/75). For the larger ZP thicknesses, the cells appeared to be more rigid and showed less apparent viscoelastic behavior. As h=R, i.e., the case of the homogeneous model, it exhibited approximately elastic behavior (Figure 6). (**B**,**C**) The variation in the micropipette radius and aspiration pressure. The larger micropipette radii and pressure values induced larger instantaneous deformations and more apparent viscoelastic behavior, because more cytoplasm was involved in the process. (**D**) The changes of aspiration length with the pressure. An approximately linear relationship was observed for the aspiration length vs. the pressure. The cells reacted strongly using a micropipette with a large radius under large pressure.

We further investigated the effects of the ZP thickness, the radius of micropipette, and the pressure on the aspiration length in the simulation. The results are shown in Figure 7. We tuned the ZP thickness β=h/R (*R* is the cell radius) and observed that a smaller instantaneous elastic deformation occurred for a large β with less obvious viscoelastic creep behavior. The aspiration length was also significantly influenced by the micropipette radius and pressure. The larger micropipette radii and pressure values induced larger instantaneous deformations and more apparent viscoelastic behavior, because more cytoplasm was involved in the process. The tendency of the aspiration length changing with time was not only affected by the mechanical properties of cell, but also the experimental conditions such as the micropipette radius and pressure. As we can expect, a homogeneous model exhibits approximately elastic deformation under small pressure and the micropipette radius. The aspiration length that was recorded 5 s after the instantaneous deformation increased linearly with the pressure, as shown in Figure 7D.

**Figure 8:**
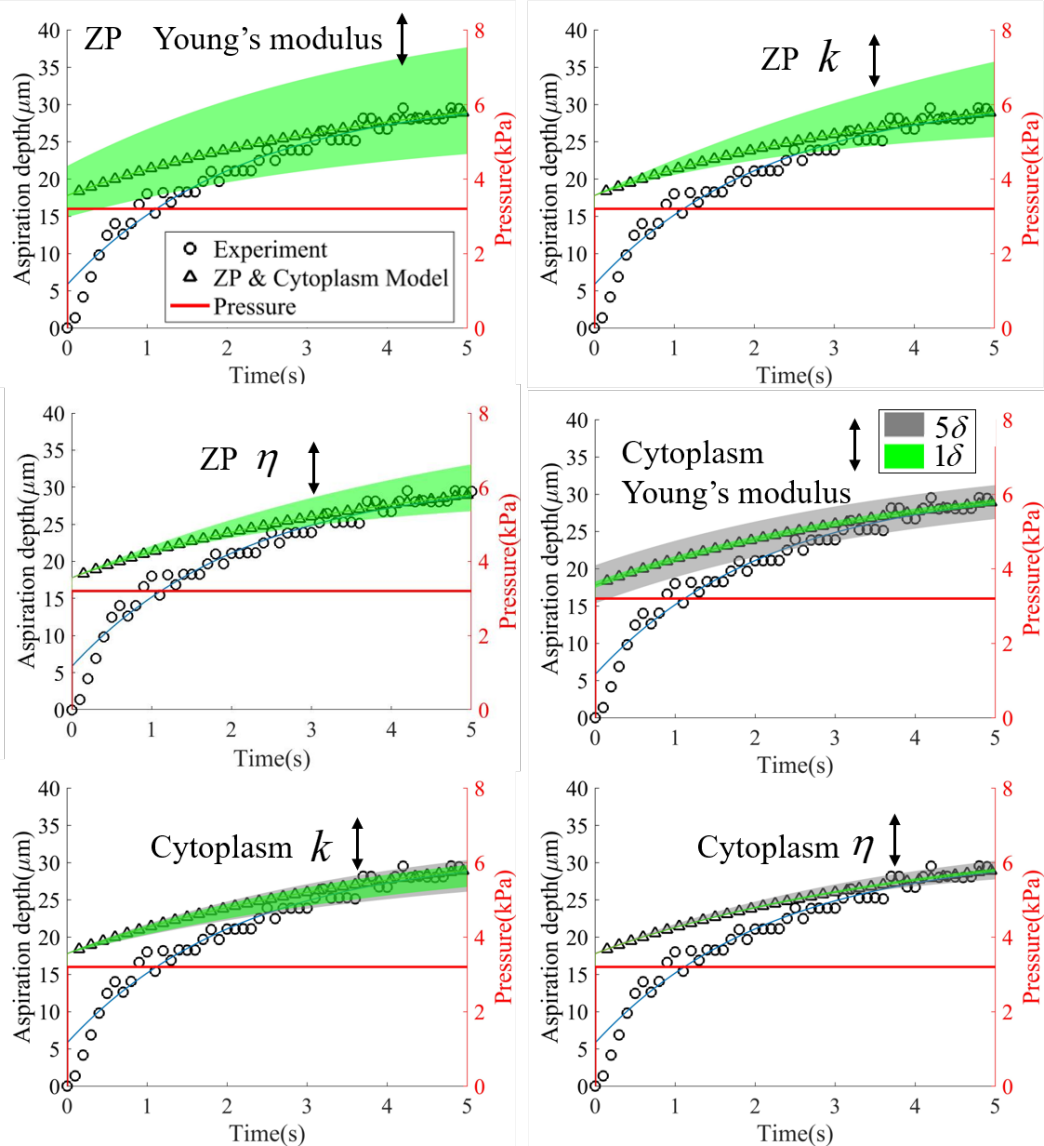
The aspiration length derived from the ZP and cytoplasm model varying with the Young’s modulus of the ZP, the *k* of the ZP, the η of the ZP, the Young’s modulus of the cytoplasm, the *k* of the cytoplasm and the η of the cytoplasm to various extents. These parameters were tuned by ±standard deviation δ (green area), as summarized in Table 1. The Young’s modulus of the ZP profoundly influenced both the instantaneous elastic deformation and final steady value, while the *k* and η of the ZP mainly affected the final steady value. Only the variation of *k* of the cytoplasm had an observable effect on the final steady value. So we changed the variation from ±1δ to ±5δ (gray area) and observed the same influence of the Young’s modulus of cytoplasm on the instantaneous elastic deformation as that of the Young’s modulus of ZP.

Figure 8 shows the effects of the Young’s modulus of the ZP, the *k* of the ZP, the η of the ZP, the Young’s modulus of the cytoplasm, the *k* of the cytoplasm and the η of the cytoplasm on the aspiration length. We tuned the parameters by ±standard deviation δ (green area). As can be seen from the figure, the Young’s modulus of the ZP profoundly influenced both the instantaneous elastic deformation and final steady value, while *k* and η of the ZP mainly affected the final steady value. In contrast, only the *k* of the cytoplasm produced an observable influence on the steady value. So we changed the variation from ±δ to ±5δ and found the same influence of the Young’s modulus of cytoplasm on the instantaneous elastic deformation as that of the Young’s modulus of ZP.

### 3.3. Validation of the Cell Model in the Microinjection Experiment

**Figure 9:**
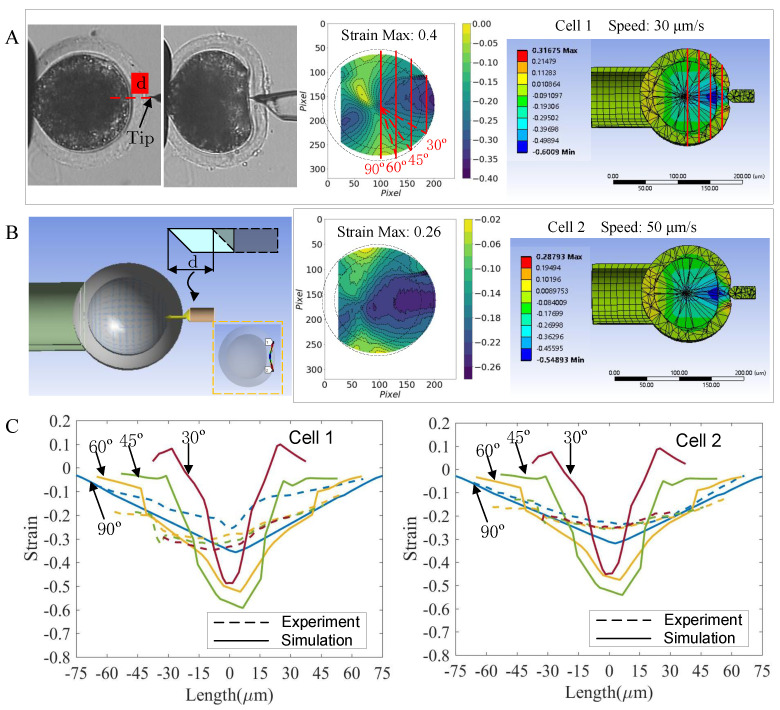
Validation of the model in the microinjection experiment. (**A**) The intracellular strain that was produced at the moment when the microinjector tip just penetrated the oocyte membrane. The microinjector moved at a speed of 30 μm/s and had the displacement of *d*μm from the start of the movement to the moment when it just penetrated the membrane (left). The microscopic images of the two frames were analyzed to obtain the experiment result (middle). The tip displacement *d* was given as the input for the model, from which the intracellular strain was generated and shown in the right image. (**B**) Moving the microinjector tip at a speed of 50 μm/s. (**C**) The comparison between the experimental and simulation results. We extracted the strain values from the four vertical lines across the half-right cell region. For the $30^{\circ }$ vertical line, the line linking the cell center and the end of the $30^{\circ }$ vertical line formed an angle of $30^{\circ }$ with the horizontal line that passed the cell center and the tip. The simulation result showed a similar strain distribution as the experimental result, but with a moderately larger maximum value.

To further validate the performance of the FEM oocyte model, we simulated the microinjection experiment and calculated the intracellular normal strain along the axis aligning with the microinjection direction from the FEM model and the recorded time-lapse microscopic images. Figure 9 shows the comparison between the experimental and simulation results of the intracellular strain of the oocytes subjected to the microinjection. From the experimental results shown in the middle images of Figure 9A,B, we observed that the lower microinjector tip speed produced a more localized strain area, but with a higher maximum strain value. This implied that the lower tip speed with prolonged movement caused the more apparent displacements of the pixel points. For our layered FEM oocyte model, the maximum strain value occurred in the neighboring region of the tip at the boundary between ZP and cytoplasm. To compare the experimental and simulation results, we acquired the values on the four vertical lines across the half-right cell region and plotted them in Figure 9C. From the two sets of lines (solid line: simulation; dotted line: Experiment), we observed a similar pattern of strain distribution, i.e., the strain in the central region (x=0 μm) was larger than that in the periphery region. This might be because the movement of the microinjector occurred along the central axis of the cell. We also found that the FEM oocyte generated a moderately large maximum strain (doubled) compared to the experimental result, which may be due to the difference in the shape and inclination angle between the simulated tip and the physical one. The luminance model for experimentally obtaining the intracellular strain caused the phenomenon that the strain was more dispersed over the cell region compared to the simulation result.

## 4. Discussion

This work presented an accurate viscoelastic oocyte model, which separately involved the role of the ZP and cytoplasm. Unlike previous work on the modeling of oocytes, our work mainly featured the following two aspects. Firstly, we considered the heterogeneity reflected by the ZP and cytoplasm, and built the oocytes as a layered spherical model, such that one layer accounted for the ZP and the other for the cytoplasm. The two layers were bonded and treated as the viscoelastic materials. Secondly, we used AFM to accurately characterize the viscoelastic properties of the ZP and cytoplasm through the approach-retraction mode and force clamping technique. The latter was used to derive the viscoelastic parameters of the sample by recording the viscoelastic creep data and fitting them with the viscoelastic spring-damping model.

To account for the variance in the cell itself, we measured multiple samples, each indented at multiple sites. The Young’s Modulus values of the cytoplasm, isolated ZP and intact ZP were 3.16 kPa, 8.77 kPa and 17.63 kPa, respectively. Due to the fluid nature of the cytoplasm, it was softer than the isolated ZP and intact ZP, which were composed of glycosylated proteins assembled into cross-linked fibrils. Our result of the isolated ZP was comparable with the results reported in [35] which used AFM to indent the isolated mature bovine ZP. A moderately larger value (doubled) was obtained for the intact ZP compared with that of the pure ZP, which was probably due to the volume effects (i.e., an intact whole spherical body vs. ZP lumps). We found that the Young’s Modulus calculated from the AFM indentation data was about 20% smaller than that estimated through micropipette aspiration in the previously published work. This might be due of the effect of the measurement scale and the possible modeling error at the macro scale.

Using the finite element analysis method, we examined the performance of the three developed viscoelastic oocyte models, namely the ZP and cytoplasm, isolated ZP and cytoplasm and homogeneous models in the simulation of the micropipette aspiration experiment. All three models led to the viscoelastic creep behavior of the cell under a step negative pressure inside the micropipette. However, the magnitude of the creeping was smaller than that observed in the experiment, which may be due to the scale difference at which the data were measured. The mechanical properties of the models were measured at the nanoscale using AFM, and the creep behavior was supposed to be less obvious at such a scale. The instantaneous elastic deformration for the experimental result was not significant, which was probably caused by the capillary pressure that gradually aspirated the cell into the micropipettte, and because it was difficult to impose a strict step pressure during the experiment. The homogeneous model and the ZP and cytoplasm model had the same Young’s Modulus of the ZP, but they exhibited different instantaneous elastic deformations, which implies the necessity of separate modeling of the ZP and the cytoplasm. Furthermore, the result of the ZP and cytoplasm agreed better with the experimental results about the aspiration length compared with the other two models. The average discrepancy in the final aspiration length (for the same time scale as the experiment) between the ZP and cytoplasm model and the experimental result when reaching the saturation was as small as 5.39%±3.42%, which also accounted for the variance in different cells.

Despite the promising results, this work has some limitations. Firstly, we measured the mechanical properties of the ZP and cytoplasm that were in good agreement with the previously recorded values and developed the viscoelastic models that were rigorously evaluated in the application of the micropipette aspiration experiment. In future work, it is desirable to apply this model in different scenarios to better understand the mechanical properties of the oocytes. Secondly, we focused here on the matured oocytes. Further models of oocytes at different development stages, such as cleavage cell or blastocysts, would lead to a better understanding of the fate of oocytes.

## 5. Conclusions

In this work, we developed an accurate viscoelastic FEM model for oocytes taking into consideration the separate roles of the zona pellucida (ZP) and cytoplasm. The viscoelastic properties of the ZP and cytoplasm were accurately characterized using AFM with the force clamping technique. The viscoelastic creep data were recorded and fitted to the Kelvin–Voigt model to derive the spring and the damping parameters. The spring and the damping parameters were used to define the shear relaxation modulus and relaxation time when developing the ZP and cytoplasm model. We examined the performance of the model in the micropipette aspiration experiment and the microinjection experiment. The results showed that this model was sufficiently accurate to reflect the true aspiration process of the oocytes under a step negative pressure. The creeping behavior of the model was in a good agreement with that shown in the experiment despite the capillary pressure and the difficulty in imposing a strict step pressure in the experiment. The model was also able to describe the deformation process of an oocyte subjected to the penetration and generated the similar intracellular strain distribution pattern as the experimental result.

## Figures and Tables

**Table 1:** A list of parameters used in cell modeling.

	Parameter	Simulation
ZP	Radius	75 μm
	Young’s Modulus	17.63 ± 5.10 kPa
	Poisson’s ratio	0.5
	*k*	0.259 ± 0.093 N/m
	η	0.290 ± 0.105 N·s/m
Isolated ZP	Thickness	20 μm
	Young’s Modulus	8.77 ± 2.38 kPa
	Poisson’s ratio	0.5
	*k*	0.216 ± 0.034 N/m
	η	0.245 ± 0.044 N·s/m
Cytoplasm	Radius	55 μm
	Young’s Modulus	3.16 ± 0.35 kPa
	Poisson’s ratio	0.5
	*k*	0.052 ± 0.005 N/m
	η	0.082 ± 0.009 N·s/m
Pipette	Inner Diameter	90 μm
	External Diameter	108 μm
	Young’s Modulus	5.5 × $10^{7}$ kPa
Contact mode	ZP and Cytoplasm	Bonded
	Pipette and ZP	Frictional
		Friction Coefficient 0.2

## Data Availability

The data that supports the findings of the study is available upon requirement.

## Supplementary Materials

The following supporting information can be downloaded at: https://www.mdpi.com/article/10.3390/mi13071087/s1.
